# Supplementary material for: The Experiences of a Complex Arts-Based Intervention for Patients with End-Stage Kidney Disease Whilst Receiving Haemodialysis: A Qualitative Process Evaluation
Source: Healthcare (Basel). 2021 Oct 18;9(10):1392. doi: 10.3390/healthcare9101392 (PMC8535624; doi:10.3390/healthcare9101392)
Supplement: Supplementary file 1 [file healthcare-09-01392-s001.zip › HCP Interview guide Process Evaluation Semi-structured interviews.pdf]

Individual Interview Guide- Healthcare Staff

The purpose of this interview is to gain a greater understanding of your experience and opinion of the arts activities and research processes that took place on the haemodialysis unit where you work. There are a few areas I would like discuss about this experience, both about the activities and different aspects of the study. If you would like to stop the interview at any time just let me know and it will be stopped. We can then start again when you are ready.

Reach

What were your first thoughts and feelings about an arts-based intervention being implemented in the haemodialysis unit where you work?
Prompts:

- What were your expectations? Did you have any concerns or worries?

Effectiveness

How do you think the arts activities impacted the patients?
Prompts:

- Did you notice a change in the patients participating in the arts activities?
- Do you think the arts activities impacted on their treatment?

Did the arts activities impact on your work or your relationship with patients?
Prompts:

- Do you feel the activities were a facilitator or barrier to communication with patients?

Adoption

How would you describe the level of engagement of patients participating in the study?

Implementation

What did you think about the arts activities and how did they impact on the clinical environment?
Prompts:

- What did you think about the way the art was facilitated?
- Were there any changes you think should be made to the activities to make them more suitable for the clinical environment?
- Were there any barriers or facilitators to implementation?

Maintenance

How would you feel about arts being provided as a resource within the haemodialysis?

Is there anything else you would like to tell me about your experience with the arts-based activities or the research process?
