# Supplementary material for: The Experiences of a Complex Arts-Based Intervention for Patients with End-Stage Kidney Disease Whilst Receiving Haemodialysis: A Qualitative Process Evaluation
Source: Healthcare (Basel). 2021 Oct 18;9(10):1392. doi: 10.3390/healthcare9101392 (PMC8535624; doi:10.3390/healthcare9101392)
Supplement: Supplementary file 1 [file healthcare-09-01392-s001.zip › Interview guide Process Evaluation Control Group.pdf]

Interview Guide- Control Group

The purpose of this interview is to gain a greater understanding of your experience and opinion of the study you participated in. There are a few areas I would like discuss about this experience. If you would like to stop the interview at any time just let me know and it will be stopped. We can then start again when you are ready.

How would you normally occupy your time while receiving haemodialysis?

Reach

What were your first thoughts and feelings about participating in the research?
Prompts:

- Have you had any previous experiences with research?
- What were your expectations? Did you have any concerns or worries?
- Did an interest in art influence your decision to participate in the study?
- How did you feel about randomisation?

Effectiveness

- Not Applicable

Adoption

Why did you remain in the study?
Prompts:

- Were there any benefits you experienced that made you continue to participate?
- Were there any difficulties that made you feel less motivated to participate?
- Do you feel your treatment/symptoms impacted on your ability to take part? Why/Why not?
- How would you feel about having the opportunity to take part in arts activities whilst receiving haemodialysis?

Implementation

How did you feel about being in the control group?
Prompts:

- Did you find it difficult to participate in research without receiving the intervention?
- How did you feel about knowing that other participants were receiving the intervention while you were not?

What did you think about the questionnaires?
Prompts:

- Do you feel they covered experiences and symptoms that are important to you?
- How did you feel while completing them? Did you find them difficult/easy to complete?
- Was there anything you found helped you complete the questionnaires ? i.e. healthcare staff/researcher facilitation/carer or family input
- Do you feel there is anything else we should be measuring?

Maintenance

Have your thoughts and feelings about art changed since taking part?
Prompts:

- How would you feel about arts being provided as a resource within the haemodialysis?

Is there anything else you would like to tell me about your experience with the research process?
