# Supplementary material for: The Experiences of a Complex Arts-Based Intervention for Patients with End-Stage Kidney Disease Whilst Receiving Haemodialysis: A Qualitative Process Evaluation
Source: Healthcare (Basel). 2021 Oct 18;9(10):1392. doi: 10.3390/healthcare9101392 (PMC8535624; doi:10.3390/healthcare9101392)
Supplement: Supplementary file 1 [file healthcare-09-01392-s001.zip › Interview guide Process Evaluation Experimental Group.pdf]

Interview Guide- Experimental Group

The purpose of this interview is to gain a greater understanding of your experience and opinion of the arts activities that you participated in during the trial. There are a few areas I would like discuss about this experience, both about the activities and different aspects of the study. If you would like to stop the interview at any time just let me know and it will be stopped. We can then start again when you are ready.

How would you normally occupy your time while receiving haemodialysis?

Reach

What were your first thoughts and feelings about participating in art while receiving haemodialysis?
Prompts:

- Did you have previous experiences/interest in art?
- What were your expectations? Did you have any concerns or worries?

Effectiveness

How did participating in art make you feel?
Prompts:

- How do you normally feel during haemodialysis sessions?
- How did you feel during the art-making sessions?
- How did you feel after the art-making sessions?
- Did you interest in/experience of art participation change at all during the trial?
- Do you feel participating in the arts has impacted your life in any other/ unexpected way? Communication/ Relationships/ Health behaviours/ Coping strategies/ Compliance with dialysis or other parts of the treatment
- Would you recommend arts-based activities to others?

Adoption

What made you want to take part and why did you remain in the study?
Prompts:

- Were there any benefits you experienced that made you continue to participate?
- Were there any difficulties that made you feel less motivated to participate?
- Do you feel your treatment/symptoms impacted on your ability to take part? Why/Why not?

Implementation

What did you think about the arts activities?
Prompts:

- What did you think about the selection of art-making materials on offer?
- What did you think about the length of the intervention?
- What did you think about the way the art was facilitated?
- Is there anything you particularly liked about the activities?
- Were there any changes you think should be made to the activities? How can we improve this intervention?

- Was there anything you found particularly helpful to completing the activities? Facilitation/Prompts/ Interest of staff

What did you think about the questionnaires?
Prompts:

- Do you feel they covered experiences and symptoms that are important to you?
- How did you feel while completing them? Did you find them difficult/easy to complete?
- Was there anything you found helped you complete the questionnaires? i.e. healthcare staff/researcher facilitation/carer or family input
- Do you feel there is anything else we should be measuring?

Maintenance

Have your thoughts and feelings about art changed since taking part?
Prompts:

- How would you feel about arts being provided as a resource within the haemodialysis?
- Have you participated in art since the intervention? In what way?

Is there anything else you would like to tell me about your experience with the arts-based activities?
